# Eosinophilic Gastroenteritis With Ascites in a Young Adult: A Diagnostic Challenge

**DOI:** 10.7759/cureus.117543

**Published:** 2026-10-06

**Authors:** Gina Abdel-Hady, Mohsen Mohamed

**Affiliations:** 1 General Surgery, Wexham Park Hospital, Slough, GBR; 2 Gastroenterology and Hepatology, Andalusia Hospital, Jeddah, SAU

**Keywords:** eosinophilic gastroenteritis diagnosis, gastrointestinal eosinophilia, inflammatory bowel diseases (ibd), systemic steroids, unexplained ascites

## Abstract

Eosinophilic gastroenteritis (EGE) is a rare gastrointestinal disorder characterised by eosinophilic infiltration of the gastrointestinal tract and associated with peripheral eosinophilia. Clinical presentation is variable and often nonspecific, while the absence of standard diagnostic criteria can contribute to diagnostic challenges. We present the case of a 24-year-old male with a two-year history of recurrent nonspecific gastrointestinal symptoms, who presented with severe abdominal pain, nausea, vomiting, and ascites. Investigations demonstrated marked peripheral eosinophilia, raised inflammatory markers, bowel wall thickening, and ascites. Histological examination of gastric and terminal ileal biopsies demonstrated gastrointestinal eosinophilic infiltration, supporting a diagnosis of EGE. The patient was treated with corticosteroids and dietary modification, with rapid resolution of symptoms and complete resolution of ascites at one-month follow-up.

This case highlights the importance of considering EGE in young patients presenting with recurrent or persistent gastrointestinal symptoms, particularly when accompanied by peripheral eosinophilia and unexplained ascites. It also emphasises the importance of reconsidering an initial diagnosis when symptoms do not follow the expected course of a self-limiting illness and maintaining a broad differential diagnosis to facilitate timely investigation and management.

## Introduction

Eosinophilic gastroenteritis (EGE) is a rare gastrointestinal (GI) condition with unclear aetiology, characterised by histological eosinophilic infiltration of the stomach, small intestines, or a combination of different organs [1]. It presents with unspecific symptoms, most commonly abdominal pain, changes to bowel habits, nausea, vomiting, and weight loss. Diagnosis can be challenging, given the uncertain presentation that could mimic other conditions such as infections, inflammatory bowel disease (IBD), or malignancy [2]. It could present equally in all age groups, with peak incidence in the third to fifth decades. It also affects both male and female populations, with a slightly higher female preponderance [3]. Currently, there is no definitive treatment plan, with management options including dietary modification, corticosteroids, biologics, immunomodulatory therapy, and surgery [4].

We report a case of EGE in a 24-year-old male with a few-year history of recurrent nonspecific GI symptoms presenting with another episode and reactive ascites. This case highlights the importance of considering EGE as a differential diagnosis in young, fit patients with persistent GI symptoms and unexplained abdominal ascites. It also highlights the role of appropriate investigation and histological assessment in establishing the diagnosis.

## Case presentation

A 24-year-old male presented to the emergency department with severe cramping abdominal pain, nausea, and vomiting. He also reported associated symptoms of weight loss, decreased oral intake, and loose stools with no blood or mucus. Notably, he had been experiencing recurrent episodes of similar symptoms over the preceding two years. This has resulted in three emergency department presentations within the previous two months. These episodes were diagnosed as viral gastroenteritis and treated symptomatically with protein pump inhibitor therapy and antiemetics. There were no associated infective symptoms or clinical features suggestive of pancreatitis or biliary pathology. He had no history of allergies and no relevant personal or familial history of atopy or autoimmune conditions. He also denied any other relevant past medical history or family history of GI conditions, specifically IBD conditions such as ulcerative colitis or Crohn’s disease. Before these episodes, he had been fit and well with no smoking or alcohol use history and no relevant travel history. 

On abdominal examination, the abdomen was slightly distended, soft, with tenderness in the left iliac fossa and umbilical region on palpation. He was clinically dehydrated, with a blood pressure of 90/70, heart rate of 110 beats per minute, dry mucous membranes, and reduced skin turgor. There was no guarding or signs of appendicitis.

Initial ultrasound scan demonstrated a mild to moderate volume of intraperitoneal fluid associated with diffuse mural thickening of the terminal ileum. Initial bloods showed raised inflammatory markers, with a CRP of 55.48 mg/L, white cell count of 12.5 × 10³/µL, neutrophils of 7.22 × 10³/µL, and eosinophils of 2.32 × 10³/µL. The remaining blood parameters, including liver and renal function, vitamin B12, coagulation screen, and autoimmune screening, were unremarkable. 

Given the presence of free peritoneal fluid, a CT thorax, abdomen, and pelvis was performed to exclude perforation. CT demonstrated moderate ascites, terminal ileal and caecal mural thickening, and associated fat stranding (Figure 1). The patient was stabilised with intravenous fluids, antiemetics, antispasmodics, and analgesia. He was then admitted under gastroenterology, with the initial working diagnosis being IBD with a high consideration of Crohn’s disease. 

**Figure 1:**
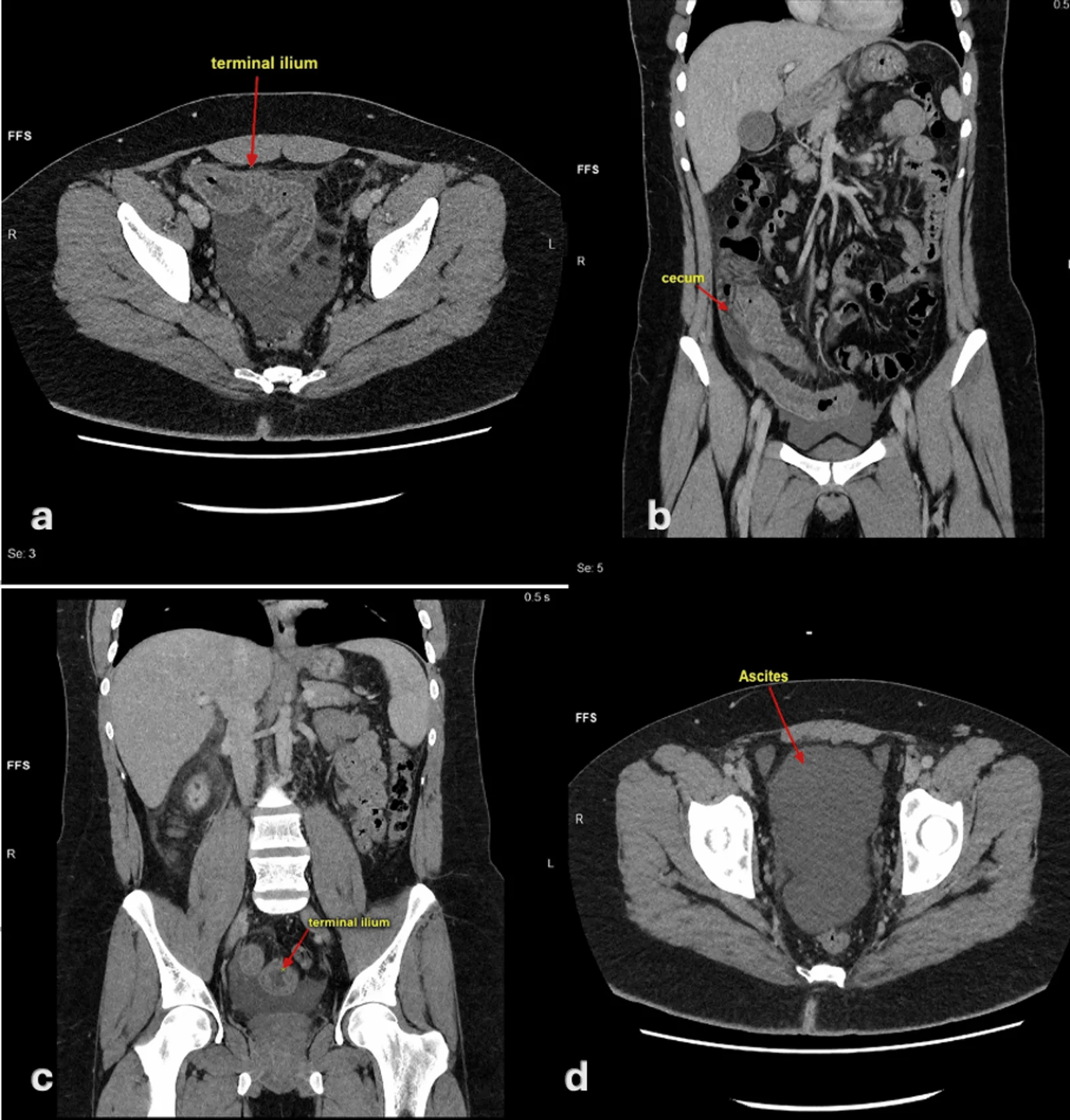
CT findings (a) Axial view demonstrating terminal ileum thickening. (b) Coronal view demonstrating caecal thickening. (c) Coronal view demonstrating terminal ileum thickening. (d) Axial view demonstrating moderate ascites.

Repeat blood tests the following day showed a white cell count of 19.8 × 10³/µL, eosinophil count of 8.46 × 10³/µL, lymphocytes of 3.7 × 10³/µL, and CRP of 17.8 mg/L. As he presented with generalised GI symptoms, further investigations included faecal calprotectin, which was elevated at 712 μg/g, and a *Helicobacter pylori* test, which was negative. Stool investigations were also performed to rule out infections, particularly parasitic, and were negative. Oesophagogastroduodenoscopy (OGD) with biopsy demonstrated mild gastropathy (Figure 2). Colonoscopy with biopsy demonstrated multiple erythematous nodular lesions with no ulceration (Figure 3). 

**Figure 2:**
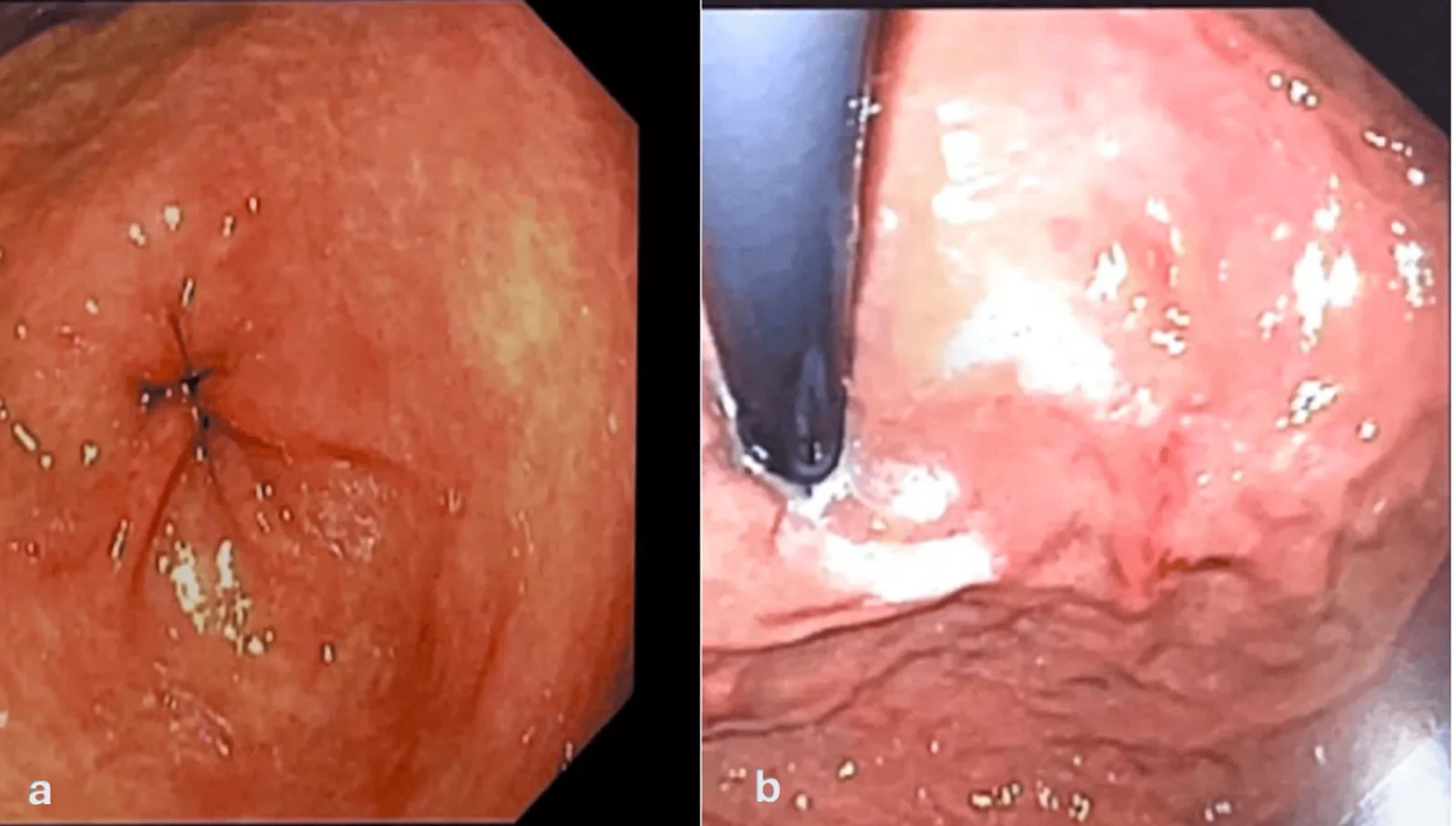
Oesophagogastroduodenoscopy findings (a) Mild gastropathy in the antrum. (b) Mild gastropathy in the stomach fundus viewed by retroflexion by the scope.

**Figure 3:**
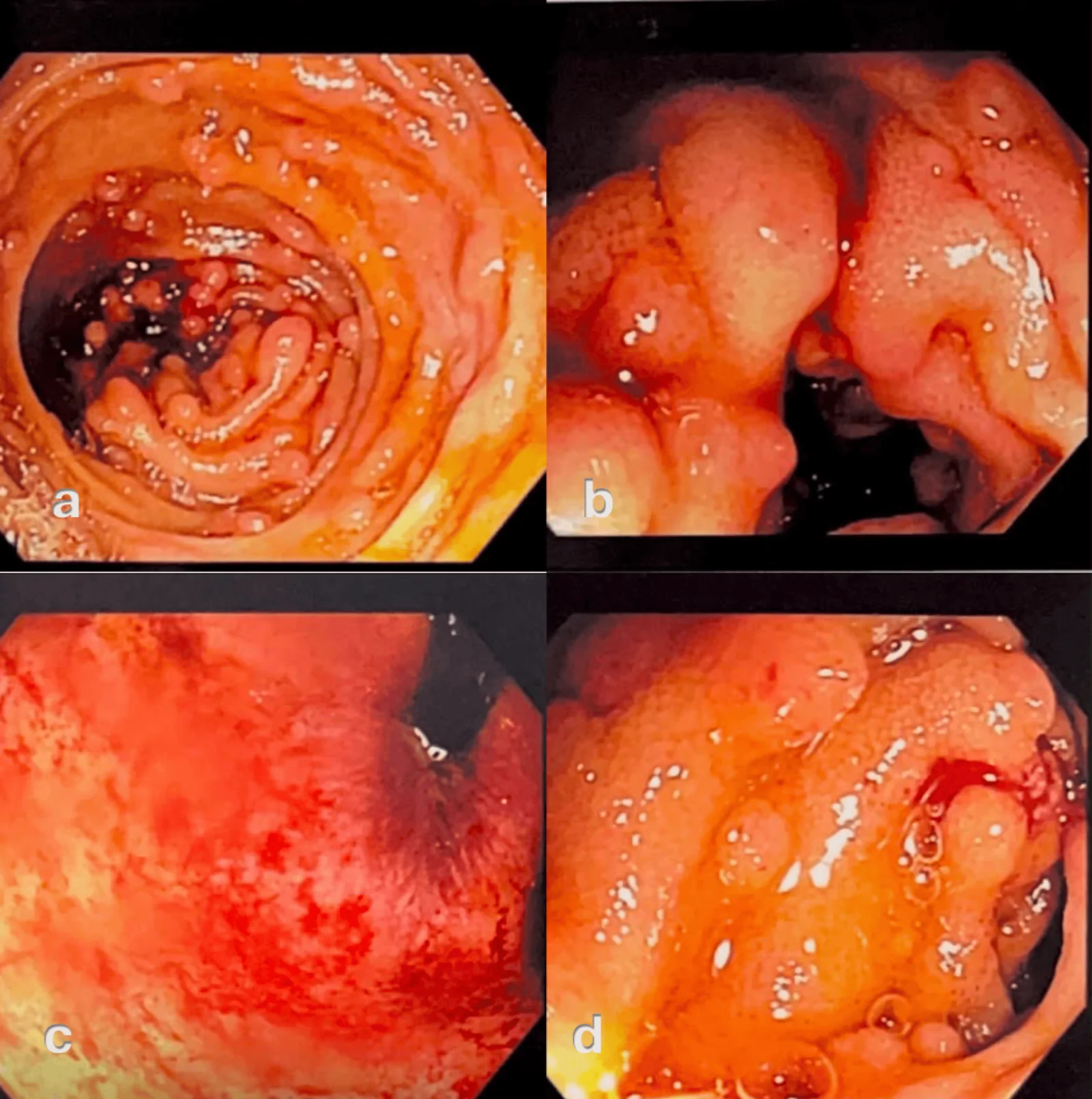
Colonoscopy findings (a) Marked erythema of terminal ileum with noted nodular lesions. (b) Marked erythema of terminal ileum with mild bleeding after taking biopsies. (c) Erythematous mucosa of anal canal on retroflexion by the scope. (d) Closer image of the marked erythema of terminal ileum mucosa with nodular lesions.

The gastric biopsies later demonstrated histological appearance of mild chronic gastritis with benign lymphoid aggregate and scattered eosinophils in the lamina propria (Figure 4). There was no evidence of glandular atrophy, intestinal metaplasia, dysplasia, or malignancy. Terminal ileum biopsies also showed marked tissue eosinophilic infiltration and a large reactive benign lymphoid aggregate with no granulomas or pseudopyloric metaplasia (Figure 5). These findings, in addition to the clinical presentation and peripheral eosinophilia, supported the diagnosis of EGE with associated ascites. 

**Figure 4:**
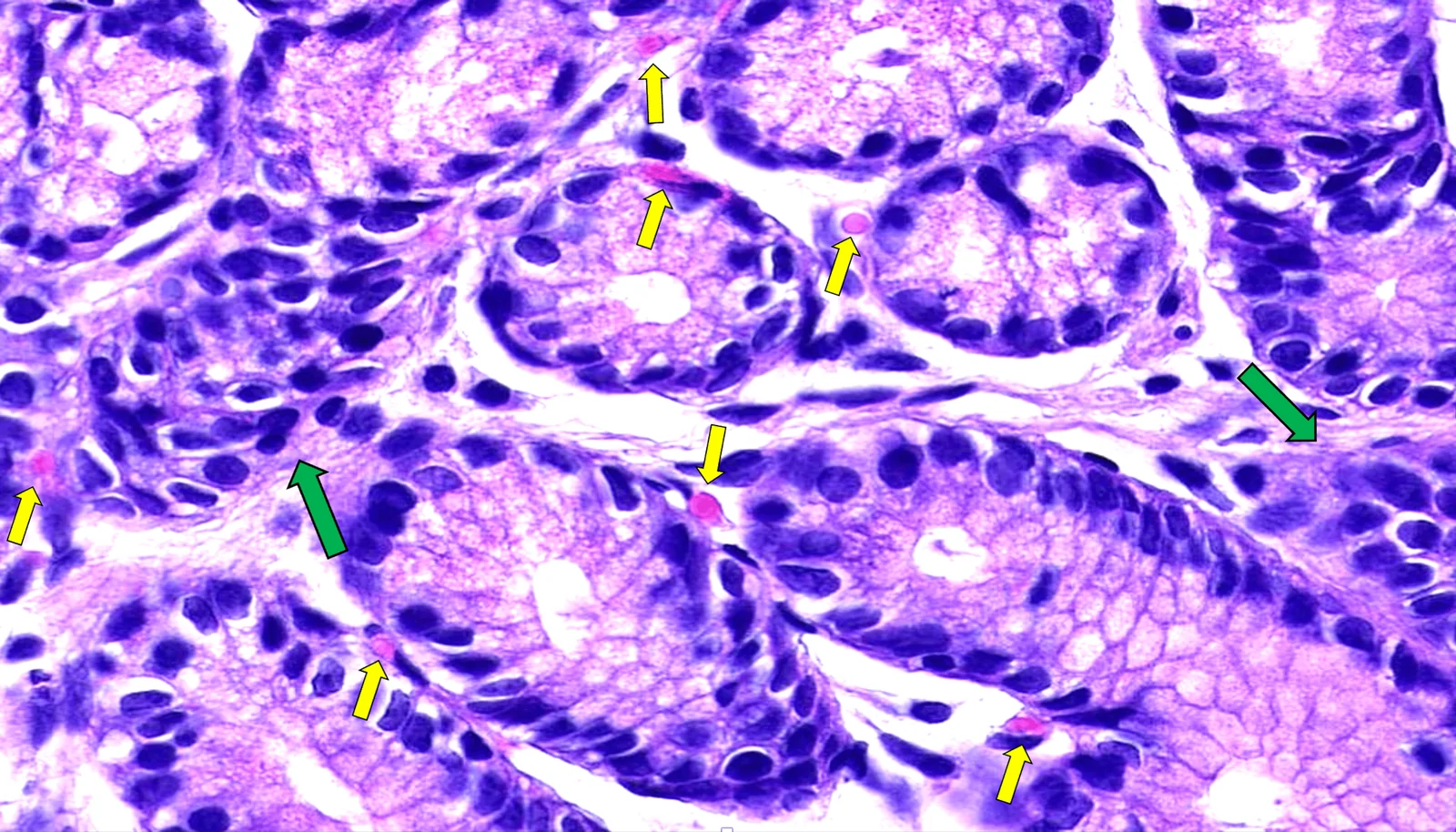
Histology slide of gastric biopsy Histology slide of the gastric lamina propria biopsy demonstrating benign lymphoid aggregate (green arrows) and scattered eosinophils in the lamina propria (yellow arrows). Staining performed: haematoxylin and eosin (H&E) staining. Magnification used: ×60.

**Figure 5:**
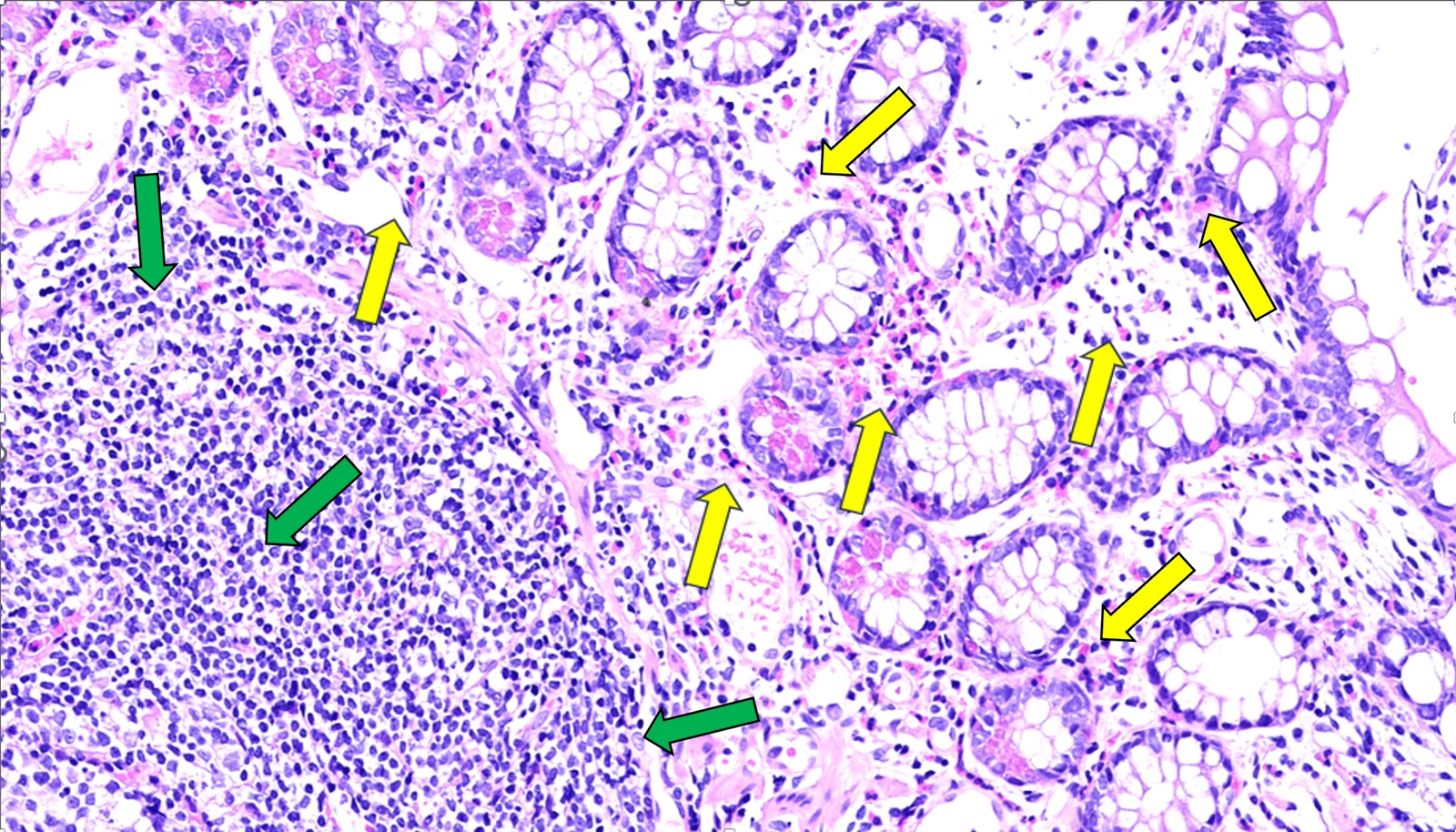
Histology slide of terminal ileum biopsy Terminal ileum mucosal biopsy demonstrating marked tissue eosinophilic infiltration (yellow arrows) and large reactive benign lymphoid aggregate (green arrows). Staining performed: haematoxylin and eosin (H&E) staining. Magnification used: ×40.

The patient was treated with intravenous hydrocortisone for three days, resulting in rapid resolution of his symptoms. He was then discharged home with a tapering course of prednisolone starting at 40 mg daily and weaned down by 5 mg weekly. Alongside, he was given dietary advice to avoid common food allergens eggs, milk, fish, wheat, nuts, and soy. He was arranged for gastroenterology outpatient follow-up after the course of steroids and a food allergen panel test to assess for possible triggers. At one-month follow-up, the patient remained in clinical remission, with complete resolution of the ascites. Abdominal examination was soft and non-tender, with no evidence of recurrent abdominal distension or other abnormal findings.

## Discussion

Eosinophilic gastroenteritis is a rare inflammatory GI condition characterised by an eosinophilic infiltrate of the stomach and/or small intestine with peripheral eosinophilia. The exact prevalence remains unknown due to the rarity of the condition and variability in reporting between studies and different countries. However, it is becoming more common and has been a recent target for research. EGE could affect a wide age range, with a peak of onset between the thirties and fifties. It previously had a slight male predominance; however, recent studies have shown that it is shifting to slight female prevalence [3].

The true pathogenesis and aetiology of the condition are still unclear. However, it is widely proposed that EGE happens due to a T-helper 2 (Th-2)-mediated allergic response to food allergens, resulting in production of interleukin-5 or mast cell degranulation. Subsequently, these result in migration of eosinophils to the GI tract [5]. EGE patients usually have a medical history of atopic conditions such as eczema, asthma, and food/medicinal hypersensitivities [6]. However, it is important to highlight that our case did not have any personal or familial history of atopy. This highlights that the absence of atopic history should not exclude EGE as a differential diagnosis. 

Clinical manifestation is generally associated with the depth of the eosinophilic infiltration and the anatomical location. The condition is usually divided into three subtypes: mucosal, muscular, and serosal [7]. The mucosal subtype usually presents with abdominal tenderness, vomiting, early satiety, bloating, diarrhoea, bleeding, malabsorption, or protein-losing enteropathy in severe cases [8]. Muscular involvement presents with muscular wall thickening and reduced gut motility, resulting in obstruction-like symptoms that include nausea, vomiting, and abdominal distension [9]. The serosal involvement is characterised by the presence of ascites in addition to symptoms of both the muscular and mucosal subtypes [10]. In our patient, the combination of GI symptoms, marked peripheral eosinophilia, bowel wall thickening, histological eosinophilic infiltration, and ascites raised suspicion for serosal involvement. However, this could not be definitively confirmed because ascitic fluid analysis was not performed.

The absence of ascitic fluid analysis represents an important limitation of this case. Ascitic paracentesis may provide additional diagnostic information in suspected serosal EGE, particularly when eosinophils predominate within the ascitic fluid [11]. In retrospect, performing an ascitic tap would have provided an opportunity to further characterise the ascites and seek additional evidence of serosal involvement. Nevertheless, the diagnosis of EGE was supported by the overall clinical, radiological, laboratory, and histological findings. The distinction is important because EGE was established histologically, whereas serosal involvement remained suspected rather than definitively demonstrated.

The prolonged history of recurrent symptoms in our patient also highlights the diagnostic challenges associated with EGE. He had experienced recurrent episodes over approximately two years and had attended the emergency department three times within two months, with each episode initially attributed to viral gastroenteritis. Diagnostic delay is well recognised in EGE, with previous studies reporting a mean delay of approximately 3.6 years, alongside substantial rates of missed and incorrect diagnoses [12]. These findings emphasise the importance of reconsidering an initial diagnosis when symptoms are recurrent, persistent, or atypical for a self-limiting GI illness. In our patient, the development of ascites and marked peripheral eosinophilia were important features that prompted further investigation beyond the initial diagnosis of gastroenteritis.

An additional diagnostic challenge in this case was the similarity between the patient’s presentation and IBD, particularly Crohn’s disease. The presence of distal ileal and caecal mural thickening, surrounding fat stranding, loose stools, abdominal pain, and an elevated faecal calprotectin initially supported a working diagnosis of Crohn’s disease. However, the marked peripheral eosinophilia and subsequent histological findings prompted consideration of EGE. Imaging findings in EGE can be nonspecific and may overlap with other inflammatory and infiltrative GI disorders, including Crohn’s disease [13]. This case therefore demonstrates the importance of interpreting bowel wall thickening and inflammatory markers in the context of the wider clinical picture, particularly when significant eosinophilia is present.

When it comes to diagnosing EGE, there are no definitive diagnostic criteria. The diagnosis is dependent on the presence of GI symptoms, histological evidence of eosinophilic infiltration, and peripheral eosinophilia in addition to the exclusion of alternative causes [14]. Imaging could identify ascites, intestinal wall thickening, and areas of involvement as well as rule out complications such as perforation or bowel obstruction [15]. Endoscopic appearances are variable and may range from erythema, ulceration, erosions, discolorations, nodules, and polyps [16]. Although biopsy and histology play an important role in diagnosing the condition [17], it is important to recognise that the presentation could be patchy and, thus, multiple biopsies may be required to confirm the diagnosis. 

Histologically, the diagnosis is characterised by pathological accumulation of eosinophils in GI tissues [18]. Thus, it is important to rule out secondary causes of eosinophilia, such as IBD, autoimmune conditions, malignancy, and hypereosinophilic syndrome (HES) [19]. These differential diagnoses need to be considered and excluded based on clinical presentation and relevant investigations. In this case, the initial radiological and biochemical findings were compatible with IBD, but the degree of peripheral eosinophilia and subsequent biopsy findings provided important evidence for EGE.

Management of EGE is generally an empirical approach guided by the severity of the presentation and anatomical involvement. Corticosteroids remain an important treatment for inducing remission, while other reported approaches include dietary modification, leukotriene receptor antagonists, mast-cell stabilisers, immunomodulators, biologic therapies, and, in selected cases, surgery [4]. Our patient demonstrated a rapid clinical response to intravenous hydrocortisone and subsequently remained in remission following a tapering course of oral prednisolone. Complete resolution of his ascites at one-month follow-up was also observed. Dietary modification was advised in this case, with avoidance of milk, eggs, wheat, nuts, fish, and soy due to EGE being highly linked to food allergens. This is known as the “6-FED” diet, which studies have shown could aid in elimination and reintroduction of food safely without triggering EGE [20].

## Conclusions

This case highlights the diagnostic challenge posed by EGE, particularly when symptoms are nonspecific and recurrent. In this patient, symptoms were attributed to viral gastroenteritis for approximately two years before the combination of ascites, marked peripheral eosinophilia, and bowel wall thickening prompted further investigation. EGE should therefore be considered in patients with persistent or recurrent GI symptoms, particularly when accompanied by peripheral eosinophilia or unexplained ascites. This case also emphasises the importance of reconsidering an initial diagnosis when the clinical course is atypical for a self-limiting illness and maintaining a broad differential diagnosis to facilitate timely investigation and management.
